# Adult ADHD in the Republic of Ireland: the evolving response

**DOI:** 10.1192/bjb.2023.77

**Published:** 2024-06

**Authors:** Shaeraine Raaj, Margo Wrigley, Richard Farrelly

**Affiliations:** 1 Connolly Hospital, Dublin, Ireland; 2 Dr Steevens’ Hospital, Dublin, Ireland; 3 Crumlin General Adult Mental Health Service, Dublin, Ireland

**Keywords:** ADHD, adult ADHD, untreated ADHD, attention-deficit hyperactivity disorders, ADHD in adults

## Abstract

Historically, attention-deficit hyperactivity disorder (ADHD) was conceptualised as a disorder of childhood that gradually improved and diminished as individuals transitioned to adulthood. Over the past decade, several studies have been published describing a cohort of adolescents with a childhood diagnosis of ADHD experiencing a continuity of ADHD symptoms into adulthood. Untreated ADHD in adults is associated with personal relationship difficulties, educational and occupational underachievement, comorbid mental health problems, substance misuse, and increased rates of road traffic accidents and criminality. These result in an increased economic burden and broader public health challenges. This review outlines the current framework and stage of development of ADHD services for adults in the Republic of Ireland.

Situated in north-west Europe, Ireland is a melting pot of cultures. The population increased from 3 million in 1950 to 5 million in 2022, with 1 million people living in Dublin, the capital city. The population of working-age people (between 18 and 65 years old) is estimated to be 3 million.^1^ The ratio between males and females is 50.1 and 49.1%.^1^ In the Republic of Ireland, there is increased recognition of adult attention-deficit hyperactivity disorder (ADHD) reported in general practice clinics and community mental health teams (CMHTs).^2,3^ It is reported that about 1.5% of adults in Ireland have ADHD, meaning about 56 000 adults are affected by the condition and meet full diagnostic criteria.^4^ The worldwide prevalence of adult ADHD is estimated at 2.8%.^5^

Several policy reforms in the past decade have led to advances in the Irish mental health system, from the initial development of the Mental Health Act 1945 to the more recently implemented Mental Health Act 2001, including legislation, policy, codes of practice and service development to improve Ireland's mental health service delivery. However, there is an opportunity for further service organisational development to improve mental healthcare delivery in the Republic of Ireland in line with government policy as outlined in *Sharing the Vision*.^6^

This review aims to outline the work in progress on developing adult ADHD services in accordance with the Health Service Executive (HSE) National Clinical Programme (NCP) for ADHD in adults and to also provide recommendations on training of psychiatrists.

## ADHD

ADHD is a common neurodevelopmental condition, the clinical characteristics of which include persistent hyperactivity, inattention and impulsivity resulting in significant psychosocial and other impairments.^7^ Historically, ADHD was first diagnosed and treated during childhood; however, recent studies recognise that the core symptoms persist into adulthood in a significant proportion of individuals.^8^ The diagnosis is recognised by the American Psychiatric Association and World Health Organization.^9^

The worldwide prevalence of childhood ADHD is estimated at 5%,^10^ and approximately 80% of children with the combined type of presentation continue to have persistent symptoms into early adulthood and meet the full diagnostic criteria.^11^ Compared with the childhood phase, the core symptoms of ADHD that progress to adulthood are predominantly inattentiveness and mild hyperactive–impulsive behaviour.^12,13^ Inattentiveness in the adult cohort is described as difficulty sustaining attention or completing a task initiated, being easily distracted, and forgetting items and appointments.^13^ Hyperactive-impulsive symptoms range from fidgeting a lot, difficulty remaining seated, restlessness, often talking excessively and ‘being very much on the go’.^14^ The features of ADHD are common in the general population, but when they are persistent and pervasive and cause problems for an individual, then a diagnosis should be considered. Functional impairment in two or more domains (social and personal relationships, education and/or occupation, and coping with everyday activities) is required to make a diagnosis in line with DSM-5 criteria.^15^

ADHD in adults is underdiagnosed in most countries, adversely affecting individuals’ overall quality of life, including increased psychological distress, academic decline, loss of employment, and increased family and economic burden.^7,10^ According to a study published in 2022, people with ADHD have lower life expectancy.^16^ Eighty per cent of adults with ADHD have at least one psychiatric comorbidity, such as depressive illness, bipolar affective disorder, anxiety disorder, sleep disorder, substance use disorder, obsessive–compulsive disorder, post-traumatic stress disorder, somatoform disorder or personality disorders.^7,17–19^ Given that parenting involves patience, attention, problem-solving and planning, a parent who is experiencing symptoms of ADHD may have marked difficulty with parenting.^20^ Parents with ADHD symptoms are reported to be more inconsistent with discipline and lack supportiveness with respect to children's emotions.^20^

Accidental death is more common among those with ADHD than those without ADHD (13.2% *v.* 4.3%, *P* = 0.052).^21^ The Swedish national registration data reported an increased in road traffic accidents and death secondary to undiagnosed and untreated ADHD.^22^ A meta-analysis in 2018 examined 32 studies and concluded that those with untreated ADHD had a 40−50% greater risk of road traffic accident injuries.^23^ Adults with ADHD have a high prevalence of comorbidities including drug and alcohol misuse.^24^

Recent research on ADHD in the prison population suggests an estimated prevalence rate of between 17 and 30% of offenders in prison meeting the diagnostic criteria for adult ADHD.^24–26^ Young and colleagues reported that nearly 25% of prisoners have ADHD, many undiagnosed or untreated.^27^ Some studies have reported that there is a possibility of treating people with ADHD to reduce impulsive behaviour and subsequently reduce potential involvement with crime and reoffending.^26,27^ It is reported that prisoners with a diagnosis of ADHD are often not treated with oral medication because of uncertainty over the reliability and validity of the ADHD diagnosis and concerns regarding potential drug misuse.^26–28^

## Adult ADHD in the Republic of Ireland

In Ireland, there is increased reporting of adult ADHD as a diagnosis in general practice CMHTs.^2,3^ Approximately 15% of working-age adults attending general adult CMHTs may have ADHD that is currently undiagnosed.^29^ Before the development of the NCP, there were no adult-specific ADHD public services available in Ireland providing assessment and treatment for ADHD in adults.^30^ In 2017, the HSE, which provides all public health services in Ireland, recognised this as a deficit and established a working group to develop a model of care for adults with ADHD. The working group included representation from ADHD Ireland, a voluntary organisation established in 1980 to provide information, resources and networking opportunities to individuals and families with ADHD. In January 2021, this NCP for adult ADHD in Ireland was formally launched by the Minister of State for Mental Health and Older People.^30^ The programme, through a multidisciplinary working group with service user representation and chaired by a clinical lead, developed a model of care covering core values and guiding principles, assessment and treatment of ADHD in line with National Institute of Health and Care Excellence (NICE) guidelines,^31^ service organisation including governance structures, the resources required, and education and training.

## NCP for adult ADHD in the Republic of Ireland

The NCP for adult ADHD recommends the development of 11 adult ADHD clinics in line with the National Model of Care to provide assessment and multi-modal treatment.^30^ The recommendation is for each adult ADHD clinic to be led by a consultant psychiatrist and have a multidisciplinary team consisting of a senior psychologist, senior occupational therapist, clinical nurse specialist and administrator.^30^ The public adult ADHD clinics would provide services to adults over 18 years old and accept referrals from general adult and child CMHTs.^30^

The development of adult ADHD clinics depends on additional funding for full implementation. Following its launch, 1.3 million euros was provided by the Department of Health to establish three pilot services. The clinical lead was asked to oversee and support the implementation of the programme nationally. The three pilot services were set up in 2022 and are now operational. These pilot services cover the following areas in Ireland:

- CHO 1: Sligo, Leitrim and Donegal
- CHO 3: Limerick, Clare and North Tipperary
- CHO 6: Dun Laoghaire, Dublin South East and Wicklow (North and South).

The 2022 Estimates Process funded a further four ADHD teams.^30^ Two teams are now operational, with the third due to start in summer 2023. Recruitment is ongoing for the fourth team. These sites are:

- CHO 4: Cork (North Lee, South Lee and North Cork) – now operational
- CHO 4: Kerry and West Cork – now operational
- CHO 7: Dublin South City, Dublin South West and Dublin West
- CHO 8: Midland counties (Laois, Offaly, Longford, Westmeath, Kildare and West Wicklow).

The national model of care for adult ADHD provides a clinical pathway including the referral procedure. The first step is for a general practitioner to refer a patient to their local general adult CMHT to be assessed for comorbid mental illness and screened for adult ADHD. Where clinical findings suggest a potential diagnosis of adult ADHD, the general adult CMHT refers the patient to the ADHD adult clinic for further assessment and management.^30^ The advantage of this cohort of patients being assessed at the secondary care level (CMHT) is that it ensures any significant mental health problems are identified and treated before the patient is referred to a tertiary-level adult ADHD clinic. The referral pathway for young people transitioning from child and adolescent mental health services (CAMHS) is modified to provide direct access from CAMHS to an adult ADHD service where ADHD is the primary problem.

This model of care ensures that adults with a potential diagnosis of ADHD can access a specialist multidisciplinary service providing an integrated, person-centred and evidence-based multi-modal response in accordance with NICE guidelines.^31^ The model includes ADHD-specific medication and non-pharmacological interventions, both group and individual. The focus is on enabling people with ADHD to manage their symptoms and develop strategies to do so, with a view to discharge within a 6−12 month period in line with the ethos of enablement.

Built into the implementation of the adult ADHD NCP is an evaluation of the three pilot sites. This is being undertaken by the University College Dublin School of Psychology over a 2 year period. It will examine the referral rate, the proportion in whom ADHD is confirmed, the demographic and psychosocial attributes of those with ADHD, interventions provided, outcomes and patient feedback on the service received. In addition, there is a second arm looking specifically at the impact of the service model on adult mental health services. The results of the evaluation will be considered, and adjustments to the model of care will be made where indicated.

## The NCP and integrated care

In line with the Sláintecare principles of integrated care for Ireland's national health service policy,^32^ the NCP has from the start worked in partnership with the voluntary sector, specifically ADHD Ireland, and with an academic partner, UCD School of Psychology. A number of projects have been completed to advocate for services for people with ADHD and to augment current public service provision. The former include a study on suicidality and repeated self-harming in people with ADHD in Ireland,^33^ and ongoing studies are in progress on ADHD in third-level students, ADHD in women and the psychosocial impacts of ADHD in adults, all focused on Ireland. The two service augmentations are an app on adult ADHD, which can be downloaded from both Apple and Google app stores, and the Understanding and Managing Adult ADHD Programme. This is a 6 week online workshop offering psychoeducation and is based on acceptance and compassion therapy. It is funded by the HSE, delivered through ADHD Ireland and led by a senior psychologist who has ADHD. Both of these initiatives are currently being formally evaluated.

In line with experience elsewhere, there is a surge of people seeking assessment for ADHD post pandemic.^34^ This can be attributed to several factors, including symptoms of ADHD being uncovered by the isolation and consequent loss of the supportive structure of work or college when working or studying at home, together with a greater public awareness of the condition. Asherson and colleagues recommended the development of an ADHD specialism within primary care, including the transfer of routine assessment, treatment and monitoring of treatment in line with NICE guidelines.^35^ This approach is currently being considered by the NCP in the context of other primary care initiatives for neurodevelopmental disorders in Ireland.

## Training of psychiatrists in ADHD in adults in Ireland

The College of Psychiatrists of Ireland (COPI) is the body recognised by the Irish Medical Council for the provision of postgraduate education in psychiatry and for the professional development of consultant psychiatrists. Whereas the assessment and treatment of ADHD is a required competency to complete training in child and adolescent psychiatry and learning disability psychiatry, it is not required for general adult psychiatry or psychiatry of old age. For this reason, the NCP has sourced training from the UK Adult ADHD Network (UKAAN) for psychiatrists, together with all other disciplines recruited to the new adult ADHD services. Furthermore, in consultation with the educational section of COPI, it secured funding to offer UKAAN training to senior registrars in psychiatry in 2020 and both senior registrars and some adult psychiatrists in 2023. Where adult ADHD services have been established, consultants offer special-interest sessions to senior registrars, and the College has approved one full-time senior registrar post in adult ADHD. The HSE, in partnership with COPI, has introduced an ASPIRE/Post CSCST Fellowship in Adult ADHD to provide the opportunity to doctors who have completed higher specialist training in psychiatry to subspecialise in adult ADHD. Trainees can also access an e-module on adult ADHD developed in 2022 by COPI.

With the expansion of adult ADHD services in Ireland, it is hoped that COPI will be able to include the assessment and treatment of ADHD in adults in the list of required competencies to complete training in general adult and old age psychiatry.

## Conclusions

Until very recently, there were no public services for adults with ADHD in Ireland. The HSE's decision to design and subsequently launch an NCP to address this significant service deficit has been a major first step in addressing this need. The provision of funding for three services in early 2021 and a further four in 2022, of which six are now operational, is an indication of significant progress. Further funding has been requested to ensure national availability. Ongoing evaluation of the programme with modification of the model of care as indicated is being undertaken. The necessity to expand in primary care in line with international experience of an unprecedented surge in referrals is also under consideration. The training of psychiatrists in this clinical area is essential, and the eventual aim is the ability to deliver this training within Ireland to all postgraduate trainees as a required competency.

## About the authors

**Shaeraine Raaj** is a consultant in the Department of Psychiatry, Connolly Hospital, Dublin, Ireland. **Margo Wrigley** is clinical lead of the Adult ADHD National Clinical Programme, Dr Steevens’ Hospital, Dublin, Ireland. **Richard Farrelly** is a consultant psychiatrist at Crumlin General Adult Mental Health Service, Dublin, Ireland.
